# Impact of uterine contractility on quality of life of women undergoing uterine fibroid embolization

**DOI:** 10.1186/s42155-019-0080-2

**Published:** 2019-11-12

**Authors:** Vinicius Adami Vayego Fornazari, Gloria Maria Martinez Salazar, Stela Adami Vayego, Thiago Franchi Nunes, Belarmino Goncalves, Jacob Szejnfeld, Claudio Emilio Bonduki, Suzan Menasce Goldman, Denis Szejnfeld

**Affiliations:** 1 Interventional Radiology and Endovascular Surgery, Universidade Federal de São Paulo (UNIFESP), Rua Napoleão de Barros, 800, Vila Clementino, São Paulo, SP 04024-002 Brazil; 2 Department of Simulation and Patient Experience, Massachusetts General Hospital, Harvard Medical School, 55 Fruit St #290, Boston, MA 02114 USA; 3 Department of Statistics, Universidade Federal do Paraná (UFPR), Rua General Carneiro, 370, Centro, Curitiba, PR 81531-990 Brazil; 4 Hospital Universitário Maria Aparecida Pedrossian, Universidade Federal do Mato Grosso do Sul (UFMS), Av. Sen. Filinto Müler, 355, Vila Ipiranga, Campo Grade, MS 79080-190 Brazil; 5 Angiography Section Clinical Interventional Radiology Department, Instituto Portugues de Oncologia (IPO-Porto), R. Dr. António Bernardino de Almeida 865, 4200-072 Porto, Portugal; 6 Department of Diagnostic Imaging, Escola Paulista de Medicina (EPM), UNIFESP, Rua Napoleão de Barros, 800, Vila Clementino, São Paulo, SP 04024-002 Brazil; 7 Outpatient Clinics of Arterial Embolization of Uterine Myoma and Cardiovascular Diseases and Thromboembolism, Gynecological Endocrinology Course, Department of Gynecology, EPM, UNIFESP, Rua Napoleão de Barros, 800, Vila Clementino, São Paulo, SP 04024-002 Brazil; 8 Department of Diagnostic Imaging, EPM, UNIFESP, Rua Napoleão de Barros, 800, Vila Clementino, São Paulo, SP 04024-002 Brazil; 9 Interventional Radiology and Endovascular Surgery, UNIFESP, Rua Napoleão de Barros, 800, Vila Clementino, São Paulo, SP 04024-002 Brazil

**Keywords:** Uterine fibroid, Leiomyoma, Dynamic MRI, Uterine peristalsis, Infertility, Quality of life, Validation studies, Questionnaire

## Abstract

**Background:**

Although changes in uterine contractility pattern after uterine fibroid embolization (UFE) has already been assessed by cine magnetic resonance imaging (MRI), their impact on quality of life outcomes has not been evaluated. The purpose of this study was to evaluate the impact of uterine contractility on the quality of life of women undergoing UFE measured by the Uterine Fibroid Symptom and Quality of Life questionnaire (UFS-QOL).

**Results:**

A total of 26 patients were included. MRI scans were acquired 30–7 days before and 6 months after UFE for all patients. The UFS-QOL was applied in person on first MRI exam day and 1 year after UFE and the outcomes were analyzed according to the groups of evolution pattern of uterine contractility: Group A: Unchanged Uterine Contractility Pattern, 38%; Group B: Favorable Modified Uterine Contractility Pattern, 50%; and Group C: Loss of Uterine Contractility, 11%. All UFE patients presented a reduction in the mean score for symptoms and increase in mean scores on quality of life. All patients in this cohort presented a reduction in mean symptom score and increase in the mean score of quality of life subscales. Group A had more relevant complaints regarding their sense of self-confidence; Group B presented worse sexual function scores before UFE, which improved after UFE compared to Group A.

**Conclusions:**

Significant improvement in symptoms, quality of life, and uterine contractility was observed after UFE in women of reproductive age with symptomatic fibroids. Functional uterine contractility seems to have a positive impact on quality of life and sexual function in this population.

**Level of evidence:**

Level 3, Non-randomized controlled cohort/follow-up study.

## Background

Symptomatic uterine leiomyoma is highly prevalent in reproductive women (Vollenhoven et al. 1990; Peregrino et al. 2017; Mas et al. 2017), with a significant impact on quality of life, affecting physical and psychological wellbeing (Spies et al. 2002). A validated quality of life questionnaire was developed to specifically assess patients with symptomatic fibroids (Uterine Fibroid UFS-Qol) and it has been widely used to evaluate changes post-fibroid treatments in several trials (Spies et al. 2002; Williams et al. 2006; Harding et al. 2008; Oliveira Brito et al. 2017; Silva et al. 2016; Beaton et al. 2000). Uterine fibroid embolization (UFE) is recognized as a Level A treatment option for the management of leiomyomas in carefully selected patients, but its use for reproductive–age women with fibroids is still controversial (Mas et al. 2017). Moreover, the impact of fibroids in infertility is not yet clear. Most patients who are willing to become pregnant choose to undergo myomectomy, yet studies have demonstrated successful pregnancies post-UFE (Pisco et al. 2017; Mohan et al. 2013). One of the hypotheses for the association between fibroids and infertility is alteration of uterine contractility, according to Kunz’s classification (Kunz et al. 1996). Uterine contractility is known to be associated with the female hormone cycle and uterine functionality (Kunz et al. 1998a). It acts to eliminate peeling endometrium during the menstrual period; influences sperm transport, nesting, embryo implantation, pregnancy maintenance during the periovulatory phase; and is implicated in dysmenorrhea (Kunz et al. 1998a; Kunz et al. 1998b; Kunz et al. 2000a; Kunz et al. 2000b; Kunz and Leyendecker 2002; Togashi 2007; Kataoka et al. 2005). It is hypothesized that some uterine disorders, such as fibroids, could alter uterine contractility and functionality (Fornazari et al. 2019; Kido et al. 2014; Kido et al. 2011; Kido et al. 2007; Koyama and Togashi 2007; Leonhardt et al. 2012; Orisaka et al. 2007). The advent of cine magnetic resonance imaging (MRI) has made it possible to quantify and evaluate uterine contractility (Kunz and Leyendecker 2002; Togashi 2007; Kataoka et al. 2005; Fornazari et al. 2019; Kido et al. 2014; Kido et al. 2011; Kido et al. 2007; Koyama and Togashi 2007; Leonhardt et al. 2012; Orisaka et al. 2007; Kido et al. 2005a; Kido et al. 2005b; Kido et al. 2006; Kido et al. 2008; Nakai et al. 2001; Nakai et al. 2003; Nakai et al. 2004; Nishino et al. 2005; Yoshino et al. 2010; Yoshino et al. 2012). Our previous study measured changes in the contractility pattern post-UFE by cine MRI, however the impact of quality of life outcomes was not evaluated (Fornazari et al. 2019). Therefore, the objective of this study was to evaluate the impact of uterine contractility on quality of life, measured by the UFS-QOL, in women undergoing uterine artery embolization for the treatment of symptomatic fibroids.

## Materials and methods

### Study design

In this Institutional Review Board (IRB)-approved, Health Insurance Portability and Accountability Act (HIPAA) compliant study, a prospective cohort of patients undergoing UFE for symptomatic fibroids, all of whom had undergone prior evaluation of uterine contractility by cine-MRI, were included. Detailed methods are reported elsewhere (Kido et al. 2005b); the inclusion, non-inclusion, and exclusion criteria are giben in Table 1 below.

**Table 1:** Inclusion, non-inclusion, and exclusion criteria

Inclusion criteria	
• Women aged 25–45 years with symptomatic fibroids and indications for embolization undergoing UFE	
• MRI performed up to 30 days before UFE and up to 6 months after UFE	
Non-inclusion criteria	
• Patients on hormonal blockade (GnRH analogues)	
• Changes in hormone profile suggestive of menopause	
• Exclusively submucosal or subserosal fibroids	
• Patients undergoing fertility therapies or assisted reproduction techniques (in vitro fertilization, intracytoplasmic sperm injection, intrauterine insemination)	
Exclusion criteria	
• Fibroids with radiological signs predictive of UFE failure, such as calcifications and absence of vascularization	
• Clinical or radiological suspicion of malignancy	
• Refusal to participate	

MRI scans were acquired 30–7 days before and 6 months after UFE, both ideally during the periovulatory cycle phase, which was estimated by adding 14 days to the first day of the last menstrual period. The Uterine Fibroid Symptom – Quality of Life (UFS-QOL) was applied in person on the first day of MRI and 1 year after UFE, during outpatient follow-up.

### UFS-QOL questionnaire

The UFS-QOL questionnaire specifically assesses severity of symptoms (8 questions) and Health-Related Quality of Life (HRQL - 29 questions) among women undergoing UFE. The HRQL scale comprises the following sub-scales: concern, activities, energy/mood, control, self-consciousness and sexual function. All items are scored on a five-point Likert scale. The higher the score on the severity subscale of the questionnaire, the greater the severity of symptoms; the lower the scores on the HRQL subscales, the poorer the quality of life (Oliveira Brito et al. 2017) (Online Resource 1).

The original UFS-QOL questionnaire was written in English (Spies et al. 2002). It has since been translated to Portuguese and this translation validated, with good internal consistency, discriminant validity, construct validity, structural validity and responsiveness, along with adequate test-retest results. It is accepted by the Society for Interventional Radiology (Beaton et al. 2000), as described in the publications of Oliveira et al. (Oliveira Brito et al. 2017) and Silva et al. (Silva et al. 2016).

All participants were able to communicate in Portuguese.

### Study measures

The image acquisition parameters consisted of obtaining scout images, followed by an SSFP (true FISP) cine MRI sequence for evaluation of contractility in the sagittal plane of the uterine cavity. This sequence was programmed to acquire a 10-mm-thick slice every 2.5 s for 4 continuous minutes, obtaining about 120 images from a single region of interest. The acquired images were viewed repetitively and consecutively at a rate of approximately 17 frames per second. Uterine contractility was defined as absent, ordered, and disordered, based on the classification of Nakai et al. (Nakai et al. 2003).

The UFS-Qol Scoring Manual was used for calculation of symptom severity. A sum score was created from the items listed below, and the formula was then used to transform the value. Higher scores are indicative of greater symptom severity, while lower scores are indicative of minimal symptom severity (Table 2).

**Table 2:** UFS-Qol formula to calculate symptom score, where higher score value indicates greater symptom severity

Scale	Sum Item Values	Lowest and Highest Possible Raw Scores	Possible Raw Score Range
Symptom Severity	Sum 1–8	8, 40	32

$$ \mathbf{Transformed}\ \mathbf{Score}=\frac{\left(\mathrm{Actual}\ \mathrm{raw}\ \mathrm{score}-\mathrm{lowest}\ \mathrm{possible}\ \mathrm{raw}\ \mathrm{score}\right)}{\mathrm{Possible}\ \mathrm{raw}\ \mathrm{score}\ \mathrm{range}}\times 100 $$

For HRQL subscales (concern, activities, energy/mood, self-conscious, and sexual function), summed scores of the items listed below were created for each individual subscale. To calculate the HRQL total score, the value of each individual subscale (not individual items) was added. Higher scores are indicative of better HRQL (high = good) (Table 3).

**Table 3:** HRQL subscales formula to calculate total score. Higher scores indicate better HRQL (high = good)

Scale	Sum Item Values	Lowest and Highest Possible Raw Scores	Possible Raw Score Range
Concern	9 + 15 + 22 + 28 + 32	5, 25	20
Activities	10 + 11 + 13 + 19 + 20 + 27 + 29	7, 35	28
Energy/mood	12 + 17 + 23 + 24 + 25 + 31 + 35	7, 35	28
Control	14 + 16 + 26 + 30 + 34	5, 25	20
Self-conscious	18 + 21 + 33	3, 15	12
Sexual function	36 + 37	2, 10	8
HRQL TOTAL	Sum of 6 Subscale Scores	29, 145	116

$$ \mathbf{Transformed}\ \mathbf{Score}=\frac{\left(\mathrm{Actual}\ \mathrm{possible}\ \mathrm{score}-\mathrm{actual}\ \mathrm{raw}\ \mathrm{score}\right)}{\mathrm{Possible}\ \mathrm{raw}\ \mathrm{score}\ \mathrm{range}}\times 100 $$

### Statistical analysis

Statistical analyses were performed using Excel and Bioestat software. The Mann-Whitney and Wilcoxon tests were used for comparisons between and within groups, respectively. A significance level of 5% was used for all tests.

## Results

### Study population

Twenty-six patients were included, with a mean age of 36 years (range 30–41 years; SD, 4 years). Of these, 14 presented with bleeding and pelvic pain, 10 with bleeding, and 3 with pain.

**Fig. 1:**
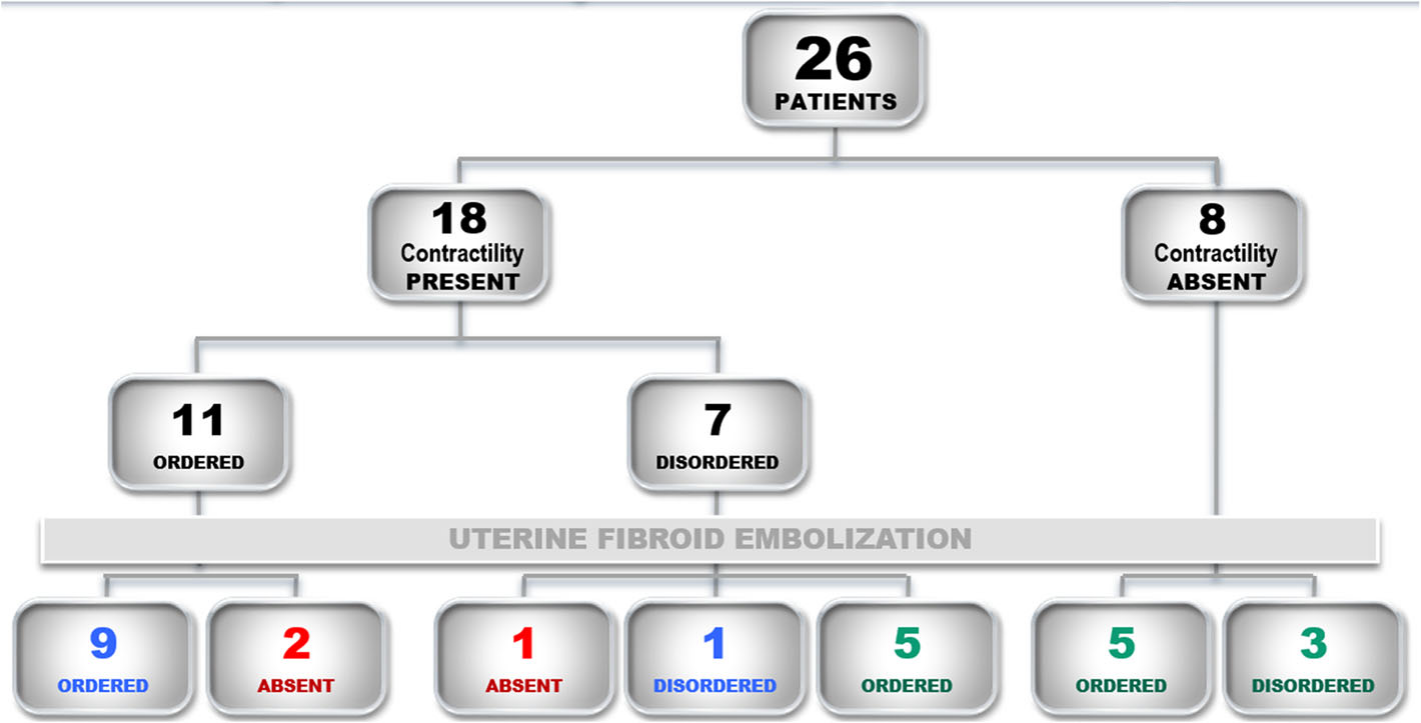
Stratification of participants by uterine contractility before and after UFE. Blue, group A; green, group B; red, group C

Three uterine contractility patterns were defined according to change in contractility from baseline after UFE: group A: unchanged uterine contractility; group B, favorably modified uterine contractility; and group C, loss of uterine contractility (Fig. 1).

Of the 26 patients included in our cohort, 10 (38%) had no change in contractility after UFE (group A), 13 (50%) had a positive change (group B), and 3 (11%) lost contractility (group C). Potential interference factors (uterine volume, necrosis pattern, fibroid localization, and index fibroid/myometrium) had no statistically significant effect (Fornazari et al. 2019).

**Fig. 2:**
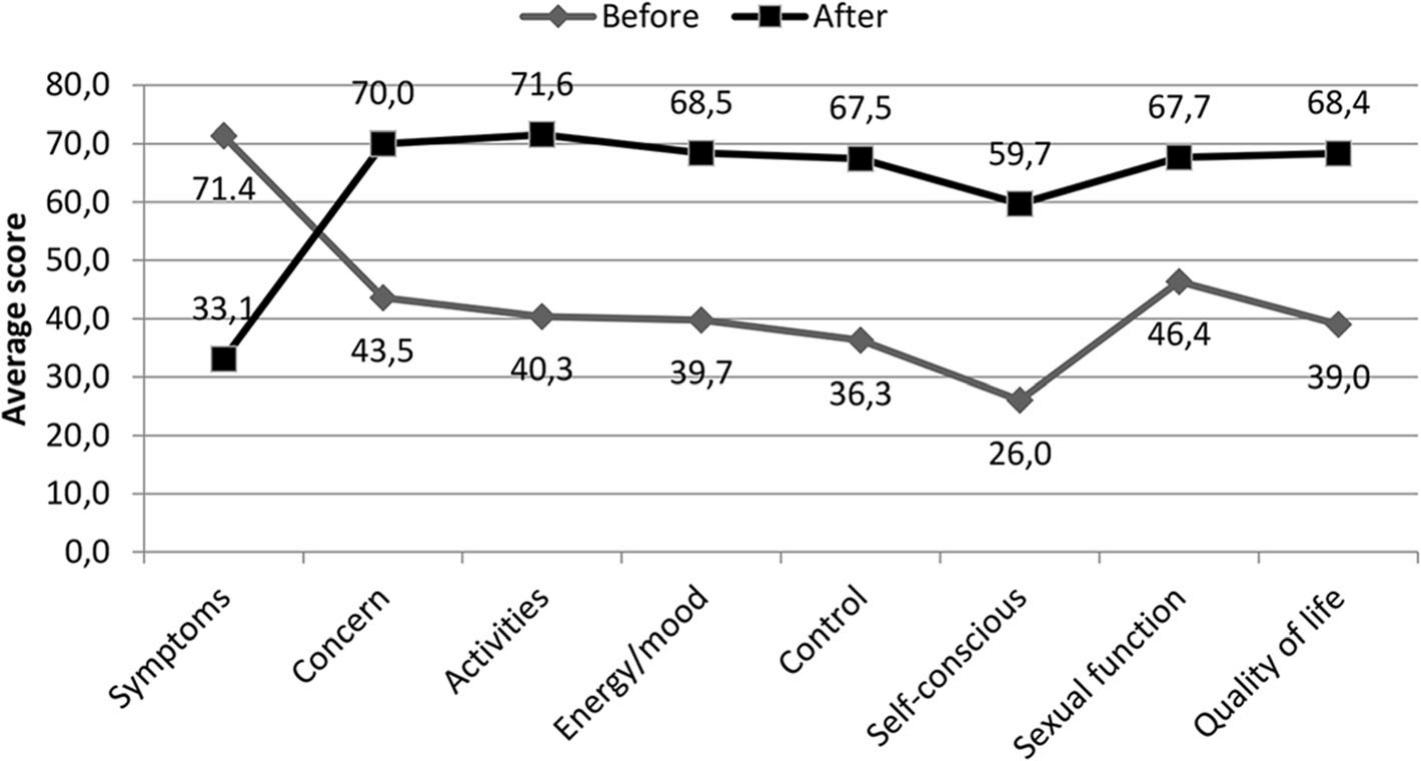
Average score obtained in the UFS-QOL questionnaire, before and after UFE

All patients in this study presented a statistically significant reduction in mean symptom score and a statistically significant increase in mean quality of life scores (worry, activity, energy, self-control, self-confidence and sexual function) (Fig. 2).

**Fig. 3:**
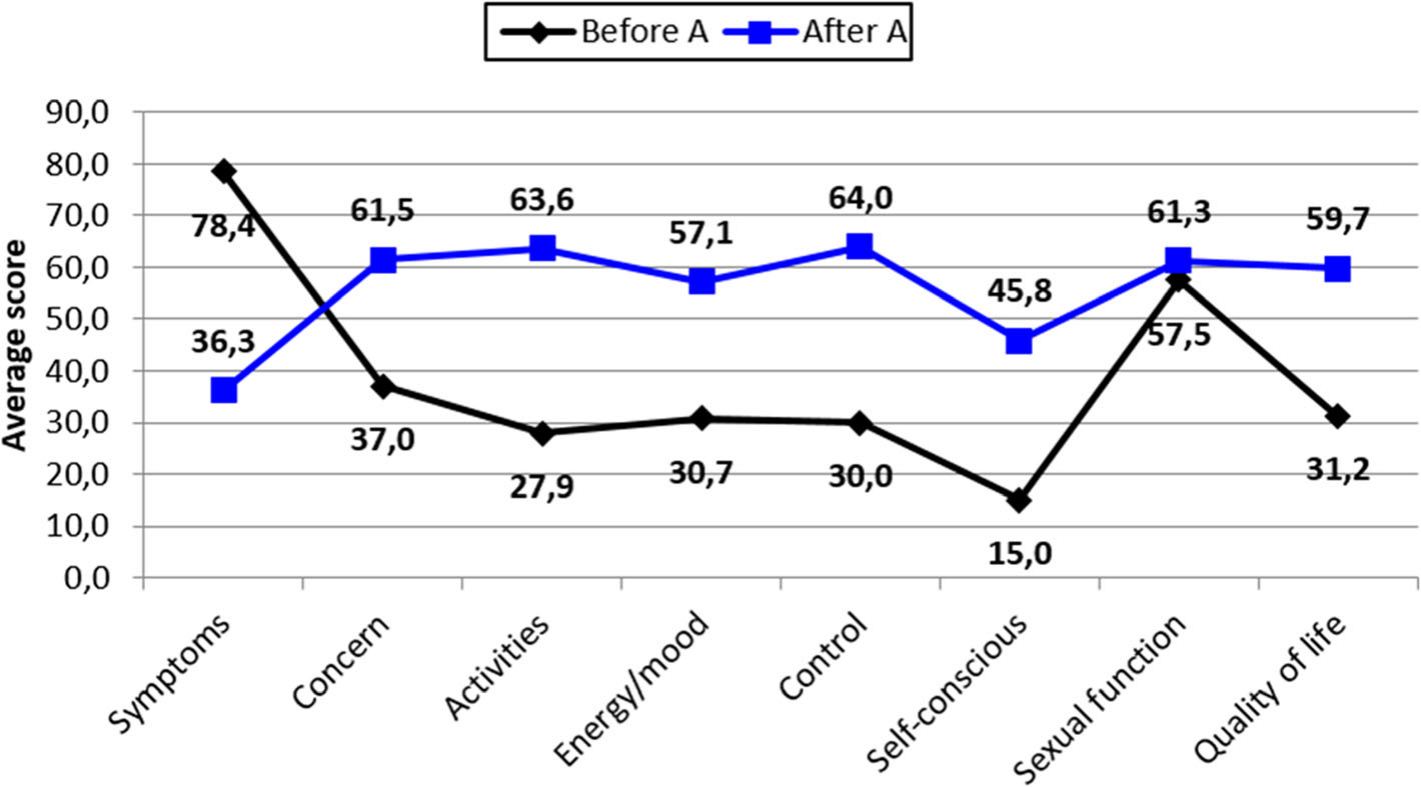
Average score obtained in the UFS-QOL questionnaire, before and after UFE, in group A

Group A patients (unchanged uterine contractility pattern) presented a statistically significant reduction in mean symptom score and increase in the mean score of quality of life subscales, except for the sexual function subscale (*p*-value = 0.3232) (Fig. 3).

**Fig. 4:**
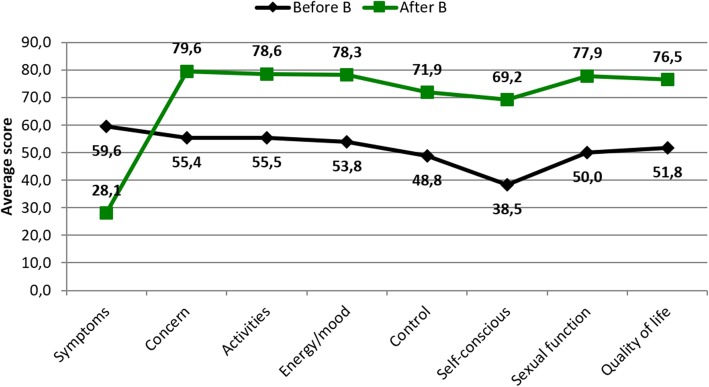
Average score obtained in the UFS-QOL questionnaire, before and after UFE, in group B

Group B (favorably modified uterine contractility pattern) showed a significant reduction in mean symptom score, and increase in mean quality of life subscale scores (Fig. 4).

As group C (loss of uterine contractility) comprised only 3 patients, no statistical analysis could be performed.

**Fig. 5:**
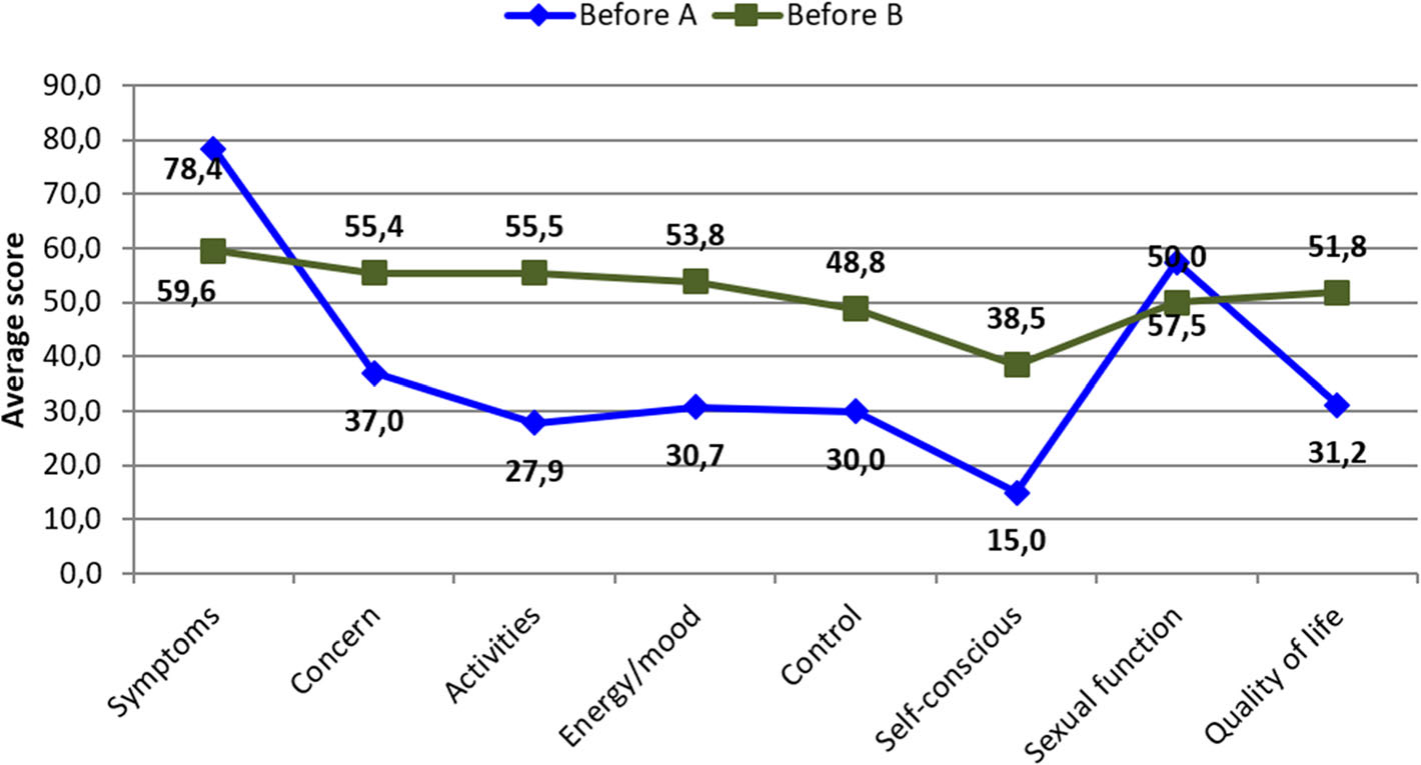
Average score obtained in the UFS-QOL questionnaire, before UAE, referring to patients in group A (blue) and group B (green)

**Fig. 6:**
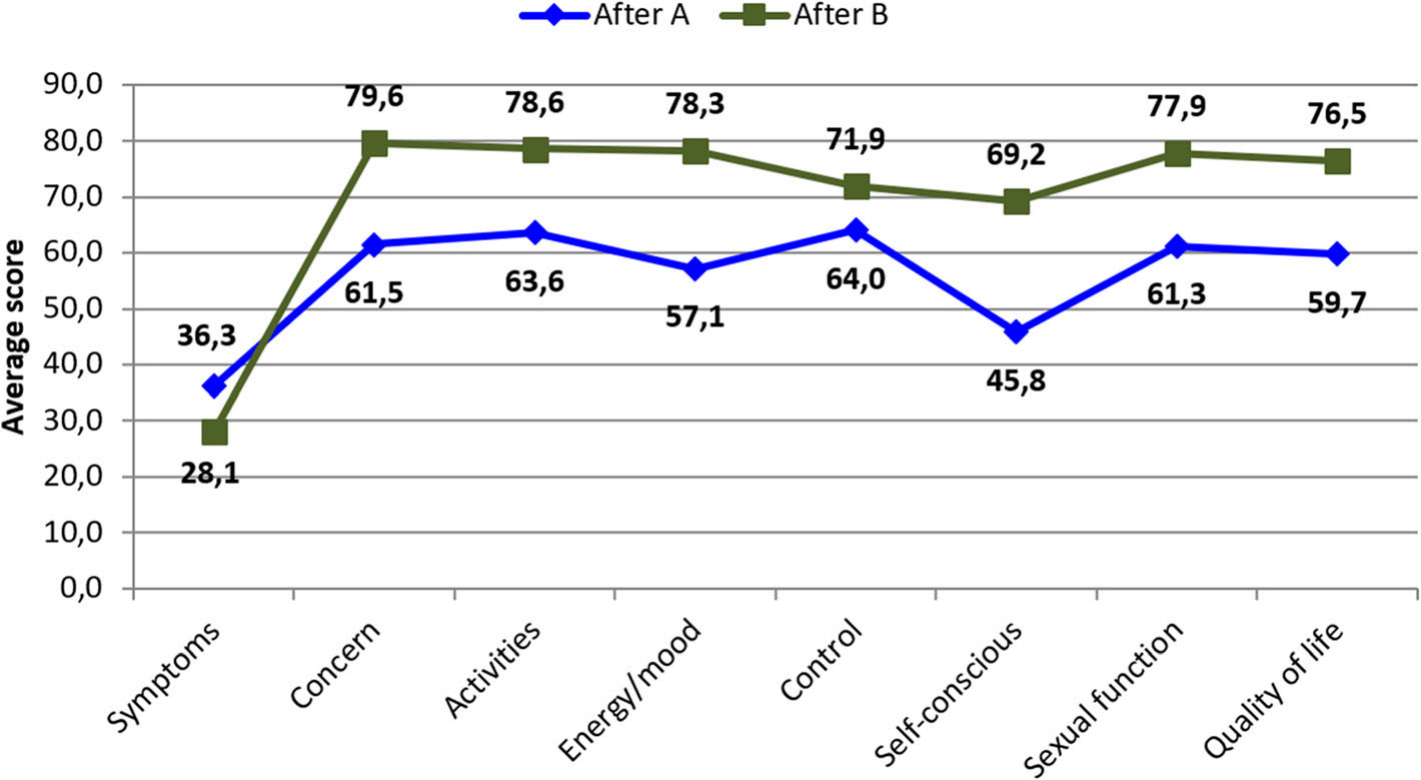
Average score obtained in the UFS-QOL questionnaire, after UFE, referring to patients in group A (blue) and group B (green)

A comparative analysis between groups A and B, before UFE, demonstrated that the average scores of activity subscales and self-confidence were significantly higher in group A (Fig. 5). After UFE, a comparative analysis between groups A and B, demonstrated significantly higher scores in group A as compared to group B (Fig. 6).

## Discussion

Most women with uterine fibroids report a negative impact of symptoms, such as abnormal uterine bleeding and pelvic pain, on their quality of life (Spies et al. 2002; Williams et al. 2006; Harding et al. 2008; Brito et al. 2014). UFE has been reported as a less morbid alternative in women who wish to preserve their uterus when hysterectomy and myomectomy are contraindicated, when fibroids are refractory to myomectomy, or when there is a high risk of conversion to hysterectomy (Spies et al. 2002; Pisco et al. 2017; Mohan et al. 2013; Fornazari et al. 2019). In this setting, UFS-QOL is a validated tool for measuring patient-reported symptoms and documenting clinical outcomes from surgical and interventional procedures (Spies et al. 2002; Williams et al. 2006; Harding et al. 2008), with good internal consistency, discriminant validity, construct validity, structural validity, test-retest similarity, and responsiveness, including in its Portuguese version (Oliveira Brito et al. 2017; Silva et al. 2016).

Our previous study began with the evaluation of uterine contractility before and after UFE in women with symptomatic fibroids using cine-MRI. Continuing this line of research, the present study aims to analyze the presentation of UFS-QoL scores among different three groups of change in uterine contractility pattern (group A: unchanged uterine contractility pattern, group B: favorable modified uterine contractility pattern, group C: loss of uterine contractility) (Fornazari et al. 2019).

Interpretation of the average USF-QOL questionnaire scores presented revealed a significant improvement in symptoms and quality of life in all patients after UFE (Fig. 4). These data before and after UFE are similar to the scores usually presented in other published series of patients undergoing UFE for symptomatic uterine fibroids (Maiara et al. 2017; Spies et al. 2005; Coyne et al. 2012). The significant improvement of uterine contractility (Fornazari et al. 2019) and simultaneous improvement of quality of life these patients experienced after UFE suggests that uterine contractility may have a positive impact on quality of life.

The three uterine contractility groups (A, B, C) did not present statistically significant demographic differences (age, pre-UFE uterine volume, post-UFE uterine volume, percentage of uterine volume reduction, total necrosis of embolized myomas and myoma-myometrium index), according to the work previously published by our group (Fornazari et al. 2019). Therefore, these factors were not associated with UFS-QOL scores.

The finding of higher average scores on the activity subscales, self-confidence, in group A (Fig. 5) demonstrates that patients in this group had more relevant complaints regarding their sense of self-confidence (conscious sensation of weight gain, size and appearance of abdomen, change in clothes when menstruating) and to activities such as fear of traveling, interference with physical activities, reduction of physical exercise, difficulty in carrying out usual activities, interference in social activities, and need for careful planning of routine activities.

Before UFE, group A presented a relatively high sexual function score in relation to other subscales; this means that sexual function was less affected in this group, and was not significantly affected after UFE (Fig. 3). In our previously published work, we did not identify statistically significant variables that could be correlated to this data (Fornazari et al. 2019). In this study, the only potentially relevant variable that could be associated with a low interference in sexual function, both before and after UFE, is the fact that this group experienced no change in uterine peristalsis.

Group B (favorably modified uterine contractility pattern) showed a significant improvement in symptoms and quality of life after UFE (Fig. 4). Sexual function scores were worse before UFE in group B and improved after UFE compared to group A.

When we analyzed groups A and B simultaneously after UFE, we found that 73% of patients (*n* = 19) presented a pattern of ordered uterine contractility and 3.8% had a disordered uterine contractility pattern (*n* = 1). Considering that all these patients experienced improvement of symptoms and quality of life after UFE, we can hypothesize that the resumption of functional uterine contractility may have a positive impact on quality of life and sexual function.

Nevertheless, our sample was small, and additional studies are required to detect the real impact of uterine contractility on fertility and quality of life.

## Conclusions

Significant improvement in symptoms, quality of life, and uterine contractility was observed after UFE in women of reproductive age with symptomatic fibroids.

Functional uterine contractility seems to have a positive impact on quality of life and sexual function in this population.

## Data Availability

The demographic data on the groups of this study are available at 10.1007/s00270-018-2053-6 Other datasets of the Uterine Fibroid Symptom and Quality of Life questionnaire (UFS-QOL) are not publicly available (due to personal information) but are available from the corresponding author (VAVF) on reasonable request.

## Abbreviations

HIPPA: Health Insurance Portability and Accountability Act
HRQL: Health-Related Quality of Life
IRB: Institutional Review Board
MRI: Magnetic resonance imaging
UFE: Uterine fibroid embolization
